# Potential habitat areas and priority protected areas of *Tilia amurensis Rupr* in China under the context of climate change

**DOI:** 10.3389/fpls.2024.1365264

**Published:** 2024-03-15

**Authors:** Fen-Guo Zhang, Sanqing Zhang, Kefan Wu, Ruxia Zhao, Guanghua Zhao, Yongji Wang

**Affiliations:** ^1^ College of Life Science, Shanxi Engineering Research Center of Microbial Application Technologies, Shanxi Normal University, Taiyuan, Shanxi, China; ^2^ Administrative Office, Shanwei Middle School, Shanwei, China

**Keywords:** *Tilia amurensis Rupr*, MaxEnt model, dominant environmental factor, Marxan model, suitable area

## Abstract

**Introduction:**

*Tilia amurensis Rupr* (*T. amurensis*) is one endangered and national class II key protected wild plant in China. It has ornamental, material, economic, edible and medicinal values. At present, the resources of *T. amurensis* are decreasing, and the prediction of the distribution of its potential habitat in China can provide a theoretical basis for the cultivation and rational management of this species.

**Methods:**

In this study, the R language was used to evaluate 358 distribution records and 38 environment variables. The MaxEnt model was used to predict the potential distribution areas of *T. amurensis* under the current and future climate scenarios. The dominant environmental factors affecting the distribution of *T. amurensis* were analyzed and the Marxan model was used to plan the priority protected areas of this species.

**Results:**

The results showed that Bio18, Slope, Elev, Bio1, Bio9 and Bio2 were the dominant environmental factors affecting the distribution of *T. amurensis*. Under the future climatic scenarios, the potential suitable areas for *T. amurensis* will mainly distribute in the Northeast China, the total suitable area will reduce compared with the current climate scenarios, and the general trend of the centroid of suitable habitat will be towards higher latitudes. The SPF value of the best plan obtained from the priority conservation area planning was 1.1, the BLM value was 127,616, and the priority conservation area was about 57.61×10^4^ km^2^. The results suggested that climate, soil and topographic factors jointly affected the potential geographical distribution of *T. amurensis*, and climate and topographic factors had greater influence than soil factors.

**Discussion:**

The total suitable area of *T. amurensis* in China under different climate scenarios in the future will decrease, so more effective protection should be actively adopted.

## 1 Introduction

*T. amurensis* is a deciduous tree of the family Tiliaceae, genus *Tilia*. It mainly grows in mixed woods and forests at altitudes of 500-1200 meters and distributes in the northeastern region of China. Over the years, the population of *T. amurensis* has declined greatly due to climatic factors and deforestation, and it has been listed as a national class II protected plant. Climatic factors are one of the most important constraints on the distribution of plant species, and as global climate problems intensify, scientists are increasingly studying climate system models. Studies have shown that climate warming and human activities, to a certain extent, not only pose a serious threat to the distribution patterns of species but even exacerbate the risk of species extinction (Urban, 2015). In addition to climatic factors, topographical and soil factors have an important influence on the distribution of plants (Chen et al., 2019). Therefore, clarifying the distribution range of suitable habitats for *T. amurensis* is important for the conservation of this species. At present, many scholars at home and abroad have conducted a great deal of research on this species in terms of breeding and afforestation (Liu et al., 2020; Li et al., 2021; Li et al., 2022b), population structural characteristics (Zhang et al., 2022), morphological features (Kim et al., 2018; Li et al., 2022a) and physiological characteristics (Sun et al., 2019). There are few studies on prediction of the potential distribution area of *T. amurensis* and the research area is small. For example, Qian Du took Heilongjiang Basin as the research area and applied MaxEnt model to simulate and predict the geographical distribution of potential species richness of major woody plants under future climate change scenarios (SSP126, SSP245, SSP585), and *T. amurensis* was one of the study objects in this study (Du, 2022).

Species Distribution Models (SDMs) correlate the geographical distribution data of species with the information on environmental variables related to the target species, and then obtain the relationship between the geographical distribution of species and environmental variables, so as to estimate the distribution of the target species by geospatial projection (Guisan and Thuiller, 2005; Wang and Ni, 2006; Guo et al., 2020). Currently, commonly used species distribution models include Classification Tree Analysis, Random Forest, Generalized Linear Models, Generalized Additive Models, Genetic Algorithms for Rule-set Prediction, and Maximum Entropy Model (Xu et al., 2015). The MaxEnt model is based on the maximum entropy, and according to the environmental variables of known pixels as the constraint condition, the possible distribution of the maximum entropy under this condition is explored, and the habitat distribution of species is predicted accordingly. MaxEnt has the advantages of low data requirements, good stability, high precision and simple operation. Its output result is a habitat suitability map with open space, and it has a built-in jackknife method to test and evaluate the significance level of individual environmental variables. Maxent has been widely used by many researchers at home and abroad to predict the potential distribution area of species. The main fields include invasive species (Ward, 2007; Zhang et al., 2023a, b), rare and endangered species (Xia et al., 2023; Li et al., 2023), and pest control (Tang et al., 2021). In this study, based on the existing distribution data of *T. amurensis*, we integrated climate, topographical and soil factors, applied the MaxEnt model to predict the potential habitat of this species in the current and future, and explored the impact of climate change on its distribution pattern. The aim of this study is to provide a basic basis for the conservation and management of the resources of this species.

Systematic conservation planning is an approach often used internationally for biodiversity conservation and the identification of priority conservation areas. The approach protects the biodiversity characteristics of an entire region through a sustainable systematic process that is purposeful, efficient in investment and of great significance to global biodiversity conservation efforts. Currently, the Marxan model is the most widely used, which is based on a simulated annealing algorithm for systematic conservation planning and can solve the minimum set problem in conservation planning (Massimiliano et al., 2016; Domisch et al., 2019; Hou et al., 2020). Some scholars have carried out relevant studies on biodiversity conservation using the Marxan model, for example, Zhang (Zhang et al., 2021) took Maoming City, Guangdong Province as the research area, applied the model to extract the elastic ecological protection space, and verified the effectiveness of the relevant results.

In conclusion, the study aimed to (1) predict the potential distribution areas of *T. amurensis* in China and compare the spatial distribution pattern under different climate scenarios; (2) analyze the main environmental factors affecting the potential geographic distribution of *T. amurensis*; and (3) delineate the priority conservation areas using the Marxan model. The results of the study can provide a scientific basis for the conservation of wild *Tilia* and its germplasm resources.

## 2 Materials and methods

### 2.1 Source of data

#### 2.1.1 Collection and collation of *T. amurensis* distribution points

**Figure 1:**
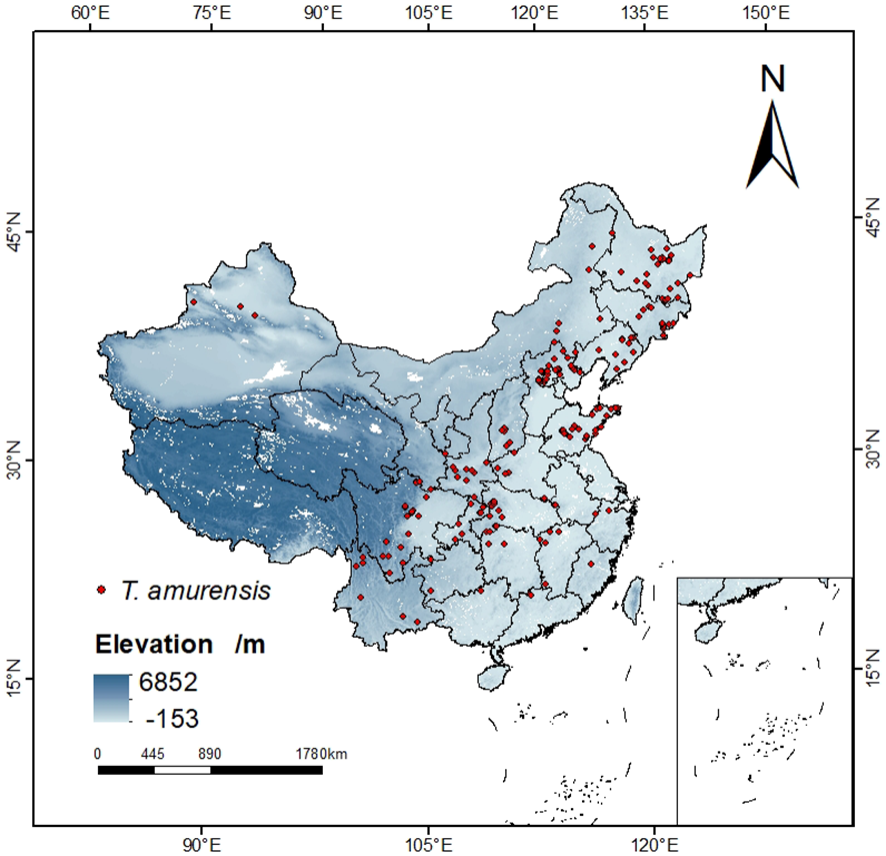
Sample distribution of *T. amurensis.*

Specimens recorded of *T. amurensis* used in this study were obtained from (1) Chinese Virtual Herbarium (CVH, http://www.cvh.ac.cn), the National Specimen Information Infrastructure (NSII, http://www.nsii.org.cn/), and other botanical herbariums; (2) Literature search. The collected distribution points of *T. amurensis* were sorted out, and 358 distribution points were obtained by manually deleting the duplicated, unclear and suspected wrong sample points. Then the geographical location information was converted into latitude and longitude coordinates of the distribution points by using the function of Baidu map “Coordinate Conversion”. In order to avoid overfitting and inaccuracy of the MaxEnt model caused by too many distribution points, this study applied the function of spThin package (Aiello-Lammens et al., 2015) in the R environment to filter the redundant distribution points in the buffer zone with a radius of 4.5km, so that only one distribution point was kept in each buffer zone to be used for the prediction work, and finally 194 distribution points were obtained. (**Figure 1**).

#### 2.1.2 Environmental variable data acquisition and preprocessing

In this study, the climate factors, soil factors and topographic factors were selected as the environmental variables based on the existing literature and the ecological characteristics of *T. amurensis*. 38 environmental variables that may affect the geographical distribution pattern of *T. amurensis* were initially considered, including 19 bioclimatic factors, 16 soil factors (0-30 cm), and 3 topographic factors. The 19 bioclimatic factors for the current (1970-2000), 2050s (2041-2060), and 2090s (2081-2100) were downloaded from the World Climate Database WorldClim (v2.1) (http://www.worldclim.org), and the spatial resolution of the data was 2.5 arc-minutes (5km), using SSP126, SSP245 and SSP585 from the Beijing Climate Center-Climate System Model-Medium Resolution (BCC-CSM2- MR),which model participated in CMIP6 (Coupled Model Intercomparison Project Phase 6) historical test simulations. Studies have shown that the simulation performance of BCC-CSM2-MR model is significantly improved compared with the earlier version BCC-CSM-1.1m (Chen et al., 2019). Soil factors and topographic factors data were obtained from the Food and Agriculture Organization of the United Nations database (http://www.fao.org/faostat/en/#data). A total of 38 environmental variables were obtained, and all environmental variable data were converted to *.asc format using ArcGIS 10.8, and it was assumed that there would be no significant changes in the soil and topographic factors in the future period (OuYang et al., 2019). Due to the collinearity problem among various environmental factors, the model prediction overfit and affect the prediction result, so the R package was used to screen all environmental variables, and the screening criteria refer to Zhang (Zhang and Liu, 2017). Spearman correlation analysis and multicollinear VIF variance expansion factor analysis were performed on the point interpolation data in R environment, and the environmental factors with correlations less than 0.7 and VIF variance expansion factor values less than 5 were initially screened. The contribution of each environmental factor in the modeling was calculated by the Jackknife method, the environmental factors with larger contributions were screened out, and then combined with the results of the previous screening, the environmental factors involved in the modeling were finally determined.

### 2.2 Model construction and evaluation

The data of 194 *T. amurensis* sample points and environment variables were imported into MaxEnt model. 75% of the sample points were randomly selected as the training set, and the remaining 25% of the sample points were used as the test set. The Jackknife test was selected to evaluate the importance of the environmental variables in the process of model construction, and to draw the Create response curve, select Random seed, the maximum number of iterations is set to 5000, select “Subsample” repetition type, repeat 10 times. The final results were output in the form of Logistic, and the other parameters were the default values of the software. The environmental factors with high contribution rates were selected based on the percentage contribution and permutation importance of each environmental factor.

The simulation results are validated in two ways, and they use the area under curve (AUC) and the size of true skill statistic (TSS) to evaluate the prediction accuracy of MaxEnt model. The AUC takes the value range of 0-1, 0.5-0.6 indicates the prediction failure, 0.6-0.7 indicates the poor prediction result, 0.7-0.8 indicates the general prediction result, 0.8-0.9 indicates the better prediction result and values between 0.9 and 1.0 indicate excellent performance. The closer the AUC value is to 1, the higher the prediction accuracy of the MaxEnt model, the more reliable the model is indicated (Dang et al., 2016). The TSS value ranges from -1 to 1, the closer the value is to 1, the better the prediction is, and 0.6 to 1 indicates a good prediction (Allouche and Kadmon, 2006).

### 2.3 MaxEnt model optimization

The regularization level of the MaxEnt model contains two parameters, regularization multiplier (RM) and feature combination (FC), which are regulated by referring to the methods of Zhao et al (Zhao et al., 2021). The MaxEnt model provides five features: linear (L), quadratic (Q), fragmentation (Hinge, H), product (P) and threshold (T). In this study, the default parameters of Maxent software are RM = 1, FC =LQHPT; in order to optimize the Maxent model, RM is set to 0. 5 ~ 4, and every time it is increased by 0. 5, a total of 8 regulated frequency doubling is made. At the same time, six combinations with one or more features were used: L; L and Q; H; L, Q and H; L, Q, H and P; and L, Q, H, P and T. According to the permutation and combination, 48 parameter combinations are calculated. In the above simulation results, if the delta.AICc value and or.10p.avg value (10% test omission rate) is lower and lower than the default parameter combination values, which indicates that the prediction results are more accurate, which leads to the optimal settings and the final model.

### 2.4 Classification of suitable areas

In this study, the Equal training sensitivity and specificity Logistic threshold is used to define the model threshold as 0.32. Based on the suitability thresholds predicted by the MaxEnt model, ArcGIS software was used to reclassify the habitat suitability index of *T. amurensis* using the natural discontinuous breakpoint grading method, which was divided into four grades: unsuitable suitable area (P<0.32), low suitable area (0.32≤P<0.5), medium suitable area (0.5≤P<0.68), high suitable area (P≥0.68).

### 2.5 Systematic conservation planning

This study is based on Marxan model for priority conservation area planning of *T. amurensis* distribution in China in the current period. The priority protection area is determined according to the following steps: (1) Planning cell and cost setting: The study area was divided into 224,767 20000m*20000m grids as planning cells, and each cell was assigned a unique ID value (Arabic numerals), and the area of each cell was set as protection cost, in square meters; (2) Construct the species distribution matrix: The potential distribution layer of *T. amurensis* in China in the current period was imported into ArcGIS, and the Zonal Statistics as Table tool of ArcGIS was used to calculate the species existence probability value (the value range is 0-1) of *T. amurensis* in each planning unit, and the species distribution matrix was constructed; (3) Determination of conservation targets: In this study, about 50% of the total number of species with a probability of species presence value greater than 0.387 was selected as the target amount for conservation; (4) Calculation of unit boundary length: ArcGIS software is applied to add plug-in ArcMarxan2.pyt to generate this data; (5) Marxan operation: The iteration times of the software were set to 1000000, and the Species Penalty Factor (SPF) and boundary length modifier (BLM) were constantly modified for optimization while the other parameters remained unchanged, with 100 operations; and (6) Visualize the priority area.

### 2.6 Validity of the results of the model simulation on the scale of the country’s administrative districts

In order to explore the validity of the model simulation results in China’s administrative region, this paper simulated the potential distribution of *T. amurensis* at the provincial and local scale in China in the current period. In this study, Heilongjiang Province and Beijing Municipality were selected as the study area. Firstly, ArcGIS software was used to extract the environmental factor data and crop vector maps of the study area. Then the MaxEnt model was used to predict the distribution of the habitable zone of *T. amurensis*, and finally the simulation results were visualized.

### 2.7 Data processing

The spThin package in R 4.2.0 software was used to process the distribution points of *T. amutilia*, the usdm package (https://cran.r-project.org/web/packages/usdm/usdm) was used to pre-process the environmental variables, the ENMeval package (Muscarella et al., 2015) was used to optimize the MaxEnt model, the SDMTool package (Naimi and Araujo, 2016)was used to calculate the position of the center of gravity in the suitable area of *T. amurensis* under the current and future climate scenarios, the geosphere package (https://cran.r-project.org/package=geosphere) was used to count the range shift distance of the centroids in different climate scenarios, and so on. The Marxan model calculated the current priority conservation areas of *T. amurensis* in China. ArcGIS 10.8 software was used for format conversion of the environmental variables, processing of the suitability classification and visualization of *T. amurensis*, and visualization of the priority protection areas. Excel software was used to organize the data and calculate the relevant area of the suitable area.

## 3 Results and analyses

### 3.1 Screening of *T. amurensis* distribution sites and environmental variables

After screening, 194 distribution point data with 17 environmental variables were finally used in this study (**Table 1**). The environmental variables include 10 bioclimatic factors (Bio 1, Bio 2, Bio 3, Bio 5, Bio 6, Bio 8, Bio 9, Bio 11, Bio 18, Bio 19), 5 soil factors (t_bs, t_cec_clay, t_oc, t_cec_soil, t_ece), 2 topographic factors (elev, slope).

**Table 1:** Environmental factors involved in modeling.

Type	Variable code	Environmental factor	Unit
Climatic factors	Bio 1	Annual Mean Temperature	°C
	Bio 2	Mean Diurnal Range	°C
	Bio 3	Isothermal property	%
	Bio 5	Maximum temperature in hottest month	°C
	Bio 6	Minimum temperature in coldest month	°C
	Bio 8	Minimum temperature in coldest month	°C
	Bio 9	Mean Temperature of Driest Quarter	°C
	Bio 11	Mean Temperature of Coldest Quarter	°C
	Bio 18	Precipitation of Warmest Quarter	mm
	Bio 19	Precipitation of Coldest Quarter	mm
Soil factors	t_bs	Topsoil Basic Saturation	%
	t_cec_clay	Topsoil CEC(clay)	cmol/kg
	t_oc	Topsoil Organic Carbon	%
	t_cec_soil	Topsoil CEC(soil)	cmol/kg
	t_ece	Topsoil Salinity(Elco)	dS/m
Topographic factors	Slope	Slope variability	%
	Elev	Altitude	m

### 3.2 Model optimization and accuracy evaluation

Based on 194 distribution points of *T. amurensis* and 17 environmental factors selected for modeling, the potential distribution area of *T. amurensis* was simulated by Maxent model. According to the method proposed by Cobos (Cobos et al., 2019), there are two models that meet the criteria, when delta.AICc=0, RM=2.5, FC type is “LQHP”, or.10p.avg=0.098, the value of the 10% training omission rate is significantly lower than the default value, and it is 40.61% lower than the default value, the model is the optimal (Muscarella et al., 2015) model.

**Figure 2:**
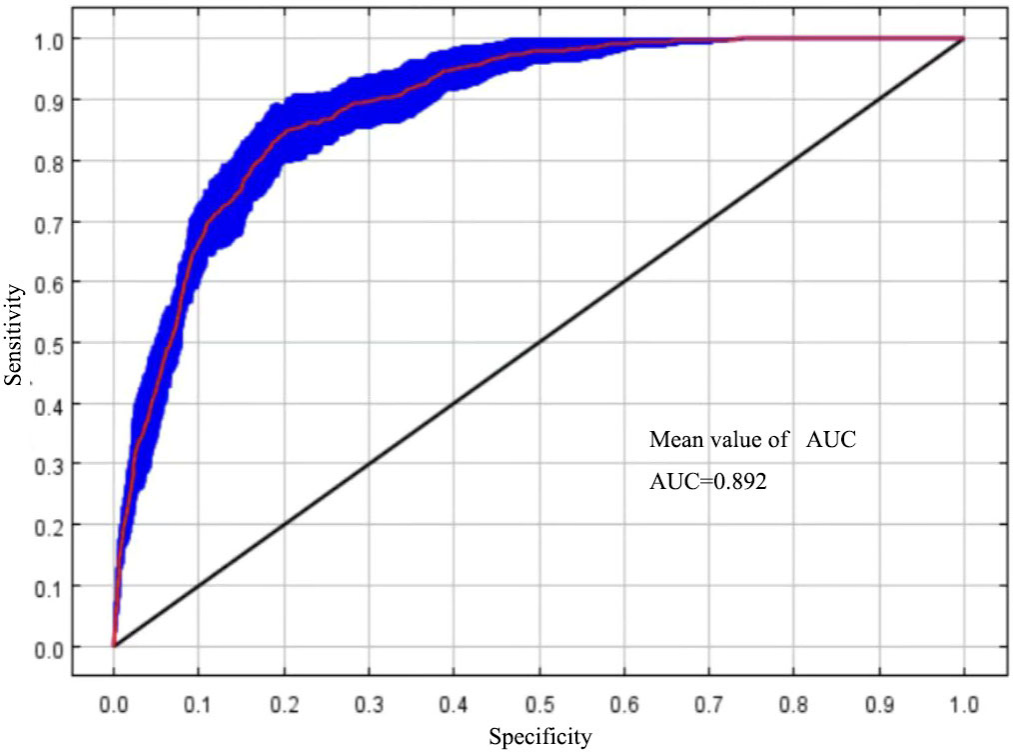
ROC curves and AUC values of MaxEnt model prediction results.

The size of the AUC value can reflect the accuracy of the model simulation effect (Han et al., 2017). In this study, MaxEnt was run under the optimal model parameter settings and cross-validated 10 times, with an average AUC = 0.892 (**Figure 2**), and the Tss value was 0.79, which indicated that the MaxEnt model can be used to simulate the prediction of the distribution of suitable areas for *T. amurensis*.

### 3.3 Analysis of dominant environmental factors

In this study, the importance of environmental variables in the process of model construction was evaluated by the Jackknife test. The results showed that: In terms of contribution rate, the top six environmental factors were Bio 18 (39.3%), Slope (14.5%), Elev (13.5%), Bio 1 (9%), Bio 9 (7.7%) and Bio 2 (5.2%), with a cumulative contribution rate of 89.2%. From the perspective of importance value, the environmental factors from high to low were Bio 18 (25.5%), Elev (20.1%), Bio 1 (19.1%), Slope (11.3%), Bio 9 (7.5%), Bio 2 (2.4%), and the cumulative importance value was up to 85.9% (**Table 2**). Among them, the contribution rate and importance value of Bio 18 are significantly higher than other environmental factors, indicating that Bio 18 was the most important environmental factor affecting the potential geographical distribution of *T. amurensis*.

**Table 2:** Contribution rate and importance of environmental variables.

Environmental variable	Permutation importance (%)	Percentage contribution (%)
Bio18	25.5	39.3
Slope	11.3	14.5
Elev	20.1	13.5
Bio1	19.1	9
Bio9	7.5	7.7
Bio2	2.4	5.2

**Figure 3:**
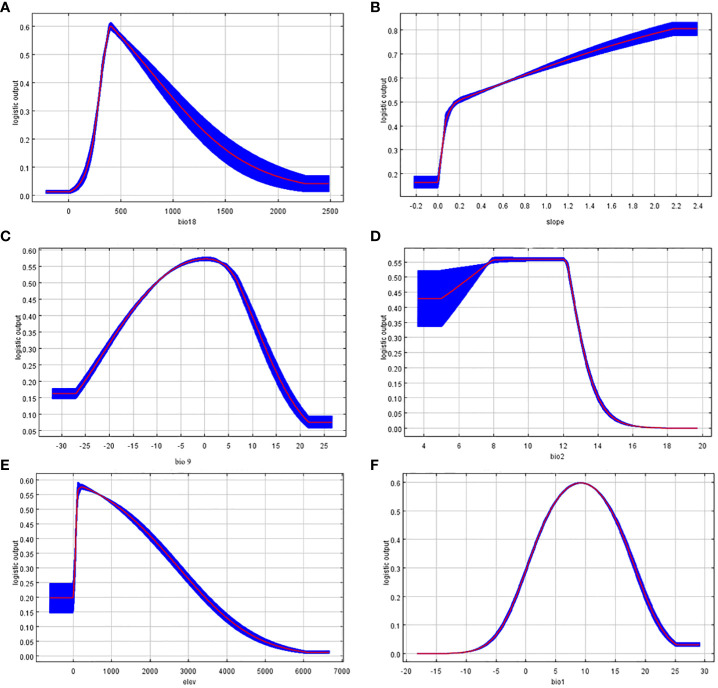
Response curve of dominant environmental factors. (**A**: bio18; **B**: slope; **C**: bio9; **D**: bio2; **E**: elev; **F**: bio1).

Environmental factors response curves reflect the dependence of suitability on variable (Zhang et al., 2020). Based on the above results, one-factor modeling was performed separately and one-factor response curves were plotted (**Figure 3**). The Bio18 value of about 407.31 is optimal for the survival of *T. amurensis*, and as this value increases, the suitability of *T. amurensis* for survival decreases. The mean temperature of driest quarter for the survival of *T. amurensis* is around 0°C.When the altitude is higher than 1200m, the suitability of *T. amurensis* decreases sharply with the increase of altitude. Slope variability of about 2.2×10^5^ is the most suitable for *T. amurensis*. The survival probability of *T. amurensis* is greatest when the Mean Diurnal Range and Annual Mean Temperature ranges are between 6°C and 12°C and around 10°C, respectively. The results are consistent with the fact that *T. amurensis* prefers cool and humid climates, does not tolerate wet and swampy areas, is cold-tolerant, born on slopes and in mixed coniferous and broad forests, and has only sporadic distribution at altitudes above 1200 m.

### 3.4 Distribution prediction and center-of-mass *range shift* of *T. amurensis* in suitable areas of China

#### 3.4.1 Current potential distribution area and size

**Figure 4:**
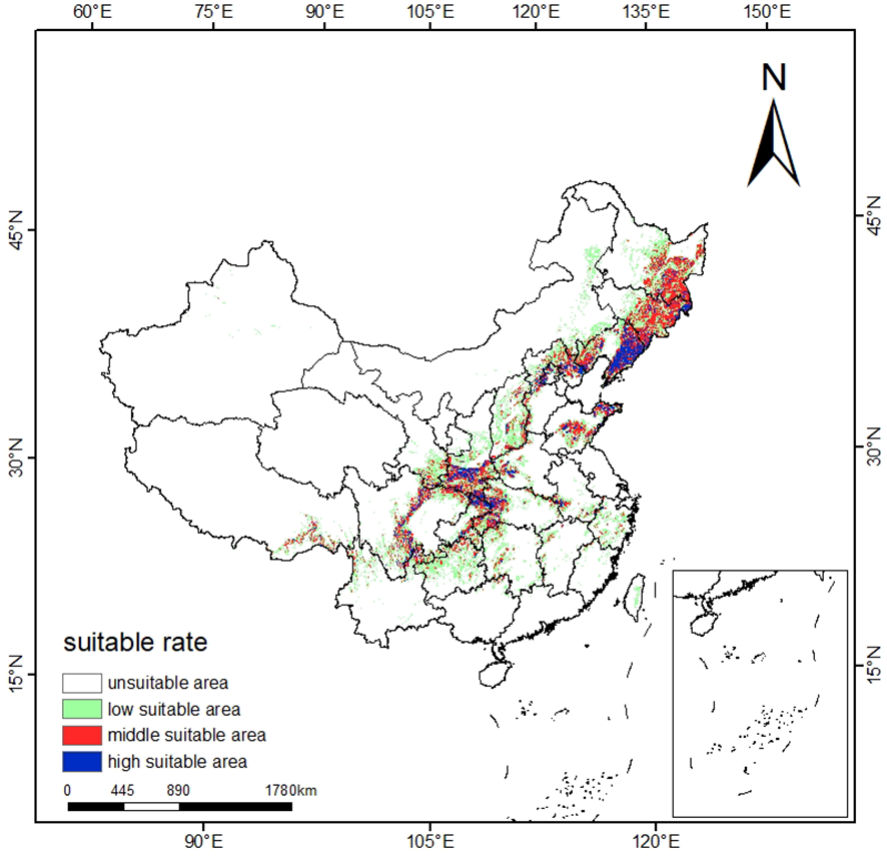
Schematic representation of the current potential distribution area of *T. amurensis* in China.

The suitable ecological distribution division of *T. amurensis* is shown in **Figure 4**, and the distribution area of each suitable area and the proportion of the total suitable area in the total study area are shown in **Table 3**. The suitable areas of *T. amurensis* in China are mainly distributed in North China, Northeast China and part of West China. The total suitable area of *T. amurensis* is about 14.15×10^4^ km^2^, accounting for 14.74% of the total area of China. Among them, the medium suitable areas are mainly distributed in Shannan and Linzhi City of Tibet, South Central Sichuan, Chongqing Municipality, Gannan Tibetan Autonomous Prefecture and Longnan Municipality of Gansu Province, South Shaanxi, West Hubei, West Henan, Southwest Anhui, Shandong Province, Hebei Province, Liaoning Province, Jilin Province, and Heilongjiang Province. The medium suitable area is about 4.9 ×10^4^ km^2^, accounting for 34.60% of the total suitable area. And the high suitable areas are mainly distributed in South Central Sichuan, South Shaanxi, West Hubei and Liaoning Province, and its area is about 1.61×10^4^ km^2^, accounting for 11.38% of the total suitable area.

**Table 3:** Suitable area of *T. amurensis* in China in the current and future periods (×10^4^ km^2^).

Scenarios	Hight suitable area	Middle suitable area	Low suitable area	Unsuitable area	Total suitable area	Proportion(%)
Current	16.1038	48.9741	76.4700	818.4521	141.5479	14.74
SSP1.26-2050s	17.0432	35.2702	55.9060	851.7805	108.2194	11.27
SSP1.26-2090s	25.0080	38.7281	62.8200	833.4440	126.5561	13.18
SSP2.45-2050s	23.1111	30.6178	56.8347	849.4344	110.5636	11.52
SSP2.45-2090s	17.6845	33.1865	56.3102	852.8188	107.1812	11.16
SSP5.85-2050s	22.2956	40.2944	68.8878	828.5221	131.4778	13.70
SSP5.85-2090s	21.7567	38.4515	59.4878	840.3040	119.6960	12.47

#### 3.4.2 Prediction of suitable area of *T. amurensis* in China under different climate scenarios

**Figure 5:**
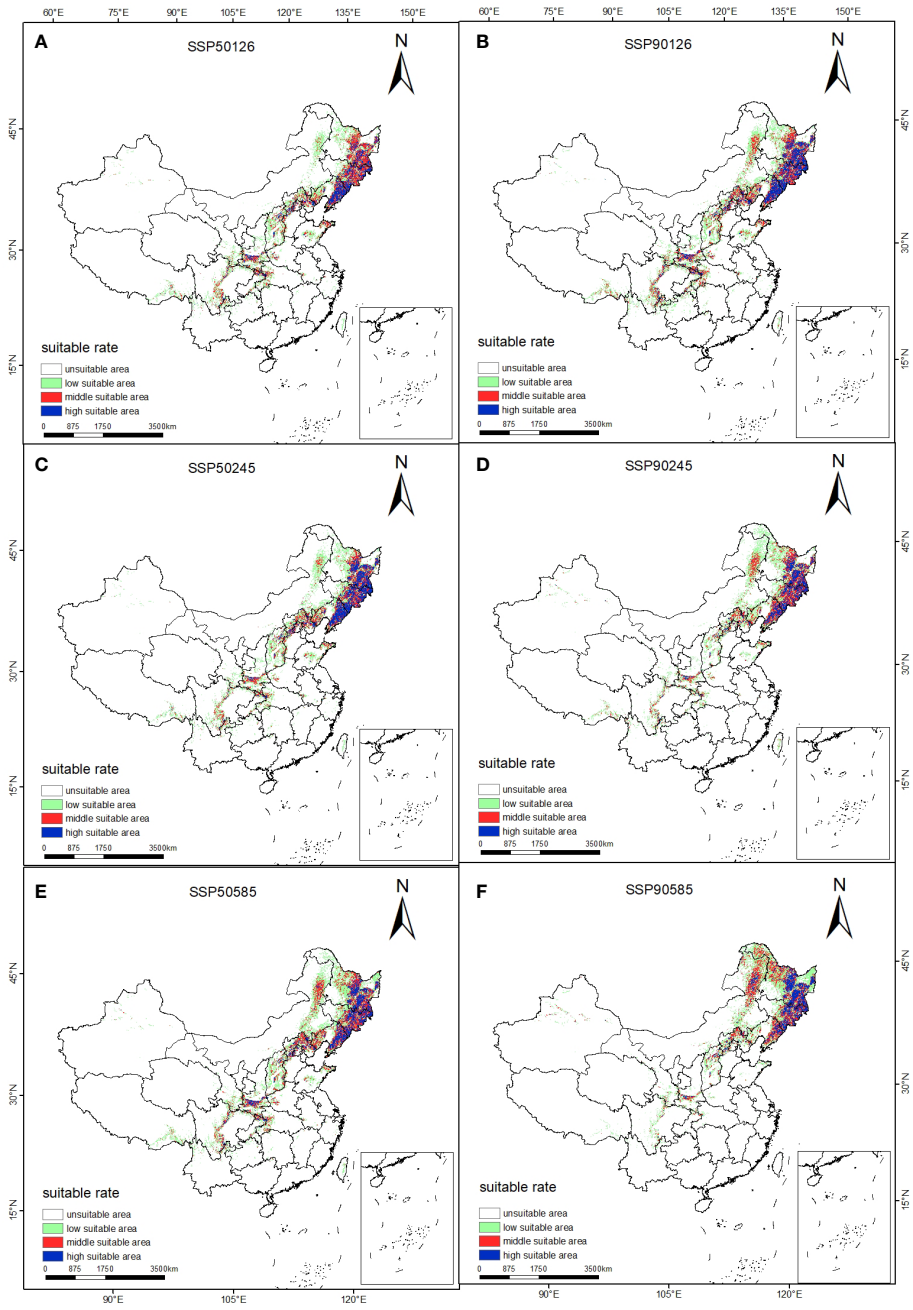
Suitable distribution for *T. amurensis* in China under different climate change scenarios in the future. (**A, B**: SSP126; **C, D**: SSP245; **E, F**: SSP585; **A, C, E**: scenarios in the 2050s; **B, D, F**: scenarios in the 2090s).

**Figure 6:**
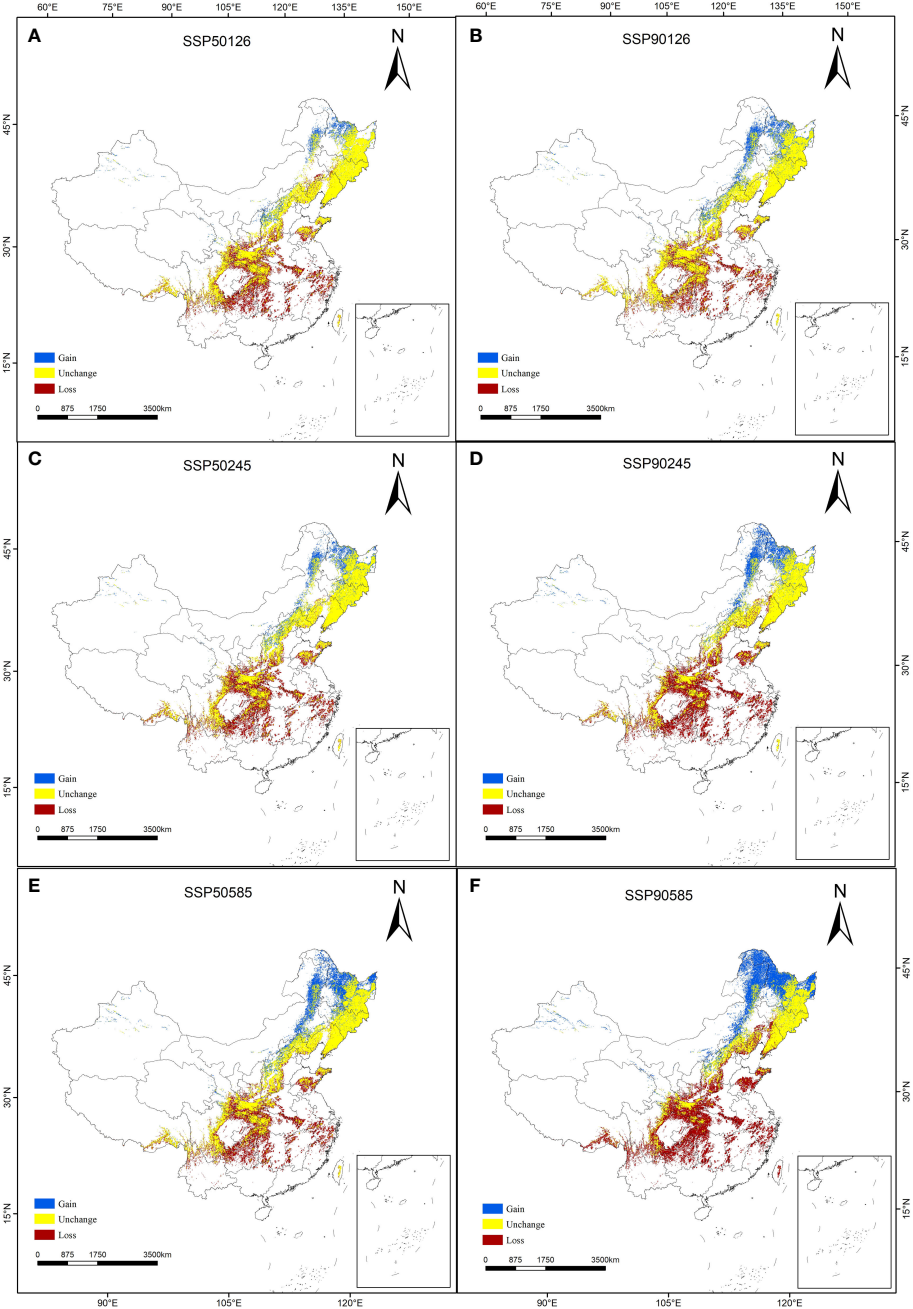
Changes in the potential habitat suitability of *T. amurensis*. (**A, B**: SSP126; **C, D**: SSP245; **E, F**: SSP585; **A, C, E**: scenarios in the 2050s; **B, D, F**: scenarios in the 2090s).

The prediction results of suitable distribution area of *T. amurensis* in China under future climate scenarios are shown in **Figure 5**. The potential suitable areas of *T. amurensis* are mainly distributed in Heilongjiang, Jilin and Liaoning Provinces, and the total suitable area will redeced under future climate scenarios. In the scenario of SSP1-2.6, the total suitable area of *T. amurensis* in 2050s and 2090s is about 10.82×10^4^ km^2^ and 12.66 ×10^4^ km^2^ respectively, decreasing by 23.55% and 10.60% compared with the current period. In the scenario of SSP2-4.5, the total suitable area of *T. amurensis* in 2050s and 2090s is about 11.06 ×10^4^ km^2^ and 10.72 ×10^4^ km^2^ respectively, decreasing by 21.89% and 24.28% compared with the current period. In the scenario of SSP1-2.6, the total suitable area of *T. amurensis* in 2050s and 2090s is about 13.15 ×10^4^ km^2^ and 11.97 ×10^4^ km^2^ respectively, decreasing by 7.11% and 15,44% compared with the current period. In the future climate scenario, unsuitability area and high suitability area will increase compared with the current period, while low suitability area, middle suitability area and total suitability area will decrease compared with the current period. The SSP5-8.5 scenario has the largest total suitable area for *T. amurensis* in 2050s, while the SSP1-2.6 scenario has the largest total suitable area in 2090s. According to **Figure 6**, it can be seen that under future climate scenarios, the suitable areas for *T. amurensis* in China will mainly increase in Heilongjiang, Jilin, and Liaoning provinces, while the lost areas will mainly distribute in central China. This trend is most obvious in the SSP90585 emission scenario.

#### 3.4.3 Changes in the center-of-mass of *T. amurensis*’s suitable area under climate change conditions

**Figure 7:**
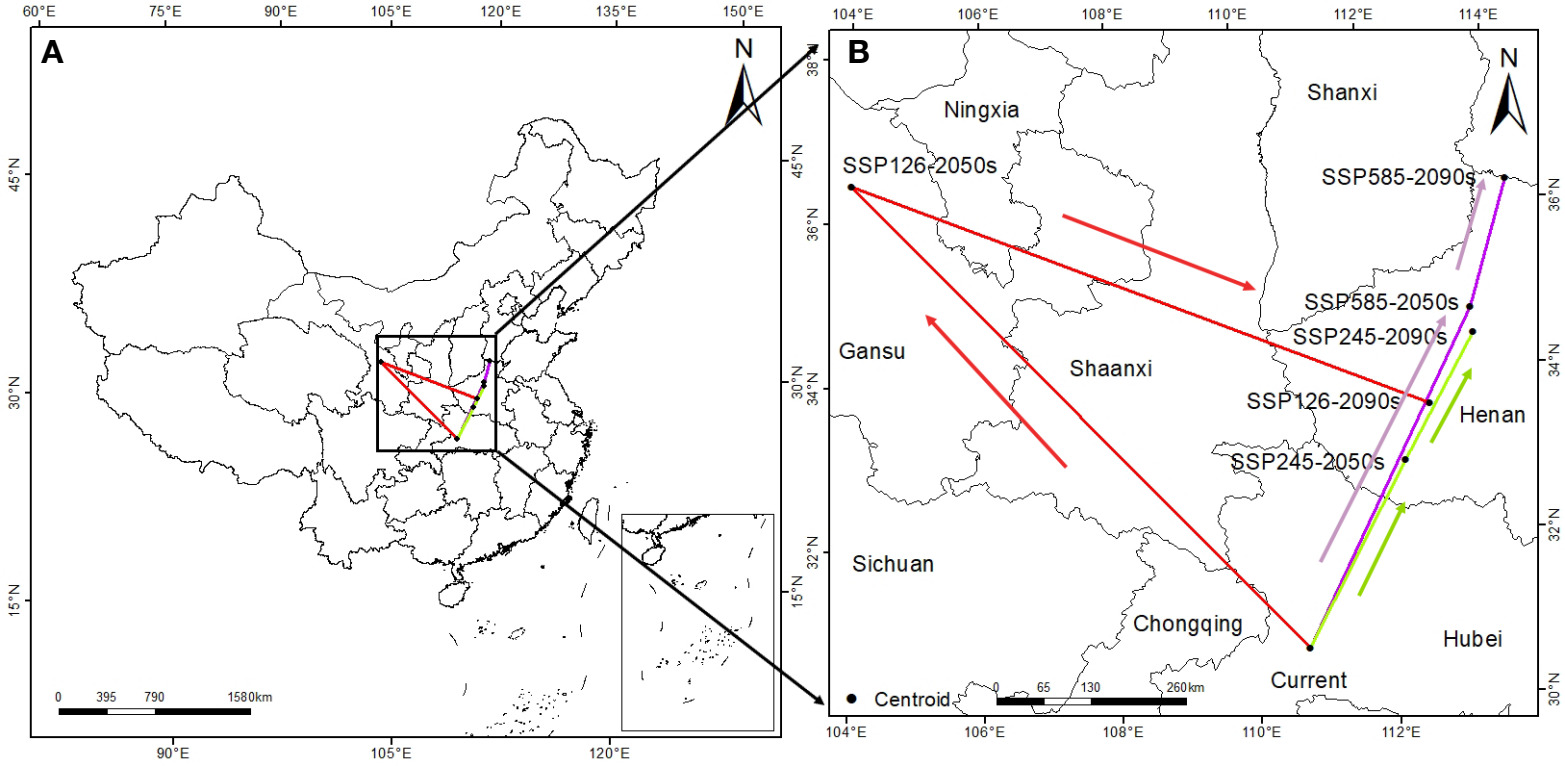
Center-of-mass range shift of suitable habitats for *T. amurensis* under different climate change scenarios. (**A**: original image; **B**: enlarged version).

To further explore the response of *T. amurensis* to future climate, this study analyzed the centroid range shift of potentially suitable habitats for *T. amurensis* under different climate scenarios in the future. The center of suitable habitat for *T. amurensis* generally tends to move to higher latitudes (**Figure 7**). The center of suitable area of *T. amurensis* in the current period is at Zigui County, Yichang City, Hubei Province. In 2050s, the center of suitable area of *T. amurensis* under the climate scenario of SSP1-2.6, SSP2-4.5 and SSP5-8.5 are located in Lanzhou City, Gansu Province, Zhoukou City, Henan Province and Pingdingshan City, Henan province, with the distances from the current center of suitable area of 895km, 289km and 515km, respectively. In 2090s, the center of suitable area of *T. amurensis* under the climate scenario of SSP1-2.6, SSP2-4.5 and SSP5-8.5 are located in Pingdingshan City, Henan province, Anyang City, Henan province and Handan City, Heubei province, with the distances from the current center of suitable area of 373km, 486km and 693km respectively.

### 3.5 Ecological characteristics of the distribution area

The results of this study showed that the habitat suitability of *T. amurensis* will decrease under different climate scenarios in the future (**Table 4**). However, compared with the current situation, the habitat suitability of *T. amurensis* under the SSP5-8.5 climate scenario will significantly reduce, and the habitat suitability is 0.45 in 2050s and 0.41in 2090s, respectively, which are 16.67% and 20.37% lower than the current situation. Precipitation of warmest quarter, annual mean temperature, and mean temperature of driest quarter were the opposite of the change in habitat suitability for *T. amurensis*, and were all higher than in the current period, but with less variation in daily differences in air temperature.

**Table 4:** Results analysis of major environmental variables.

Environmental variable	Current	SSP1-2.6		SSP-2.45		SSP5-8.5	
		2050s	2090s	2050s	2090s	2050s	2090s
Bio 18	431.84	436.23	466.83	480.71	476.64	501.70	536.49
Slope	3.65x10^5^	3.65x10^5^	3.65x10^5^	3.65x10^5^	3.65x10^5^	3.65x10^5^	3.65x10^5^
Elev	905.91	905.91	905.91	905.91	905.91	905.91	905.91
Bio 1	8.71	10.68	11.06	11.68	10.79	12.12	14.12
Bio 9	-4.44	-1.83	-1.25	-1.07	-1.89	-0.30	1.65
Bio 2	10.36	10.56	10.30	10.32	10.37	10.53	10.54
Suitability of species habitat	0.54	0.49	0.50	0.50	0.52	0.45	0.41

### 3.6 Priority protected area planning

**Figure 8:**
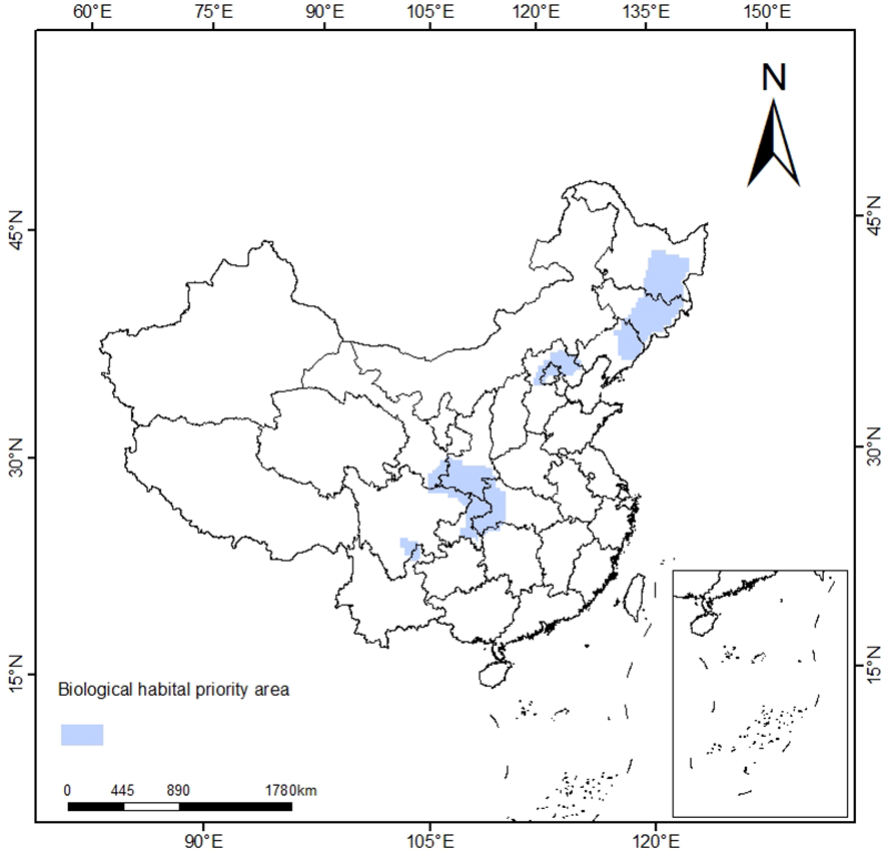
Priority Conservation Area Plan.

Through sensitivity analysis, different SPF and BLM combinations were compared, and the optimal combination was finally determined as SPF=1.1, BLM=127616, 1632 planning units were selected, and the priority protection area was about 5.76×10^4^ km^2^. The priority conservation areas of *T. amurensis* are shown in **Figure 8**, mainly distributed in Jilin, Heilongjiang, Hebei, Gansu, Shaanxi, Henan, Sichuan, Chongqing and other provinces as well as Beijing Municipality, which is basically consistent with the distribution results of *T. amurensis* in middle and high suitability areas in China as predicted by Maxent model.

### 3.7 Validity of modelling results at national, provincial and local scale

**Figure 9:**
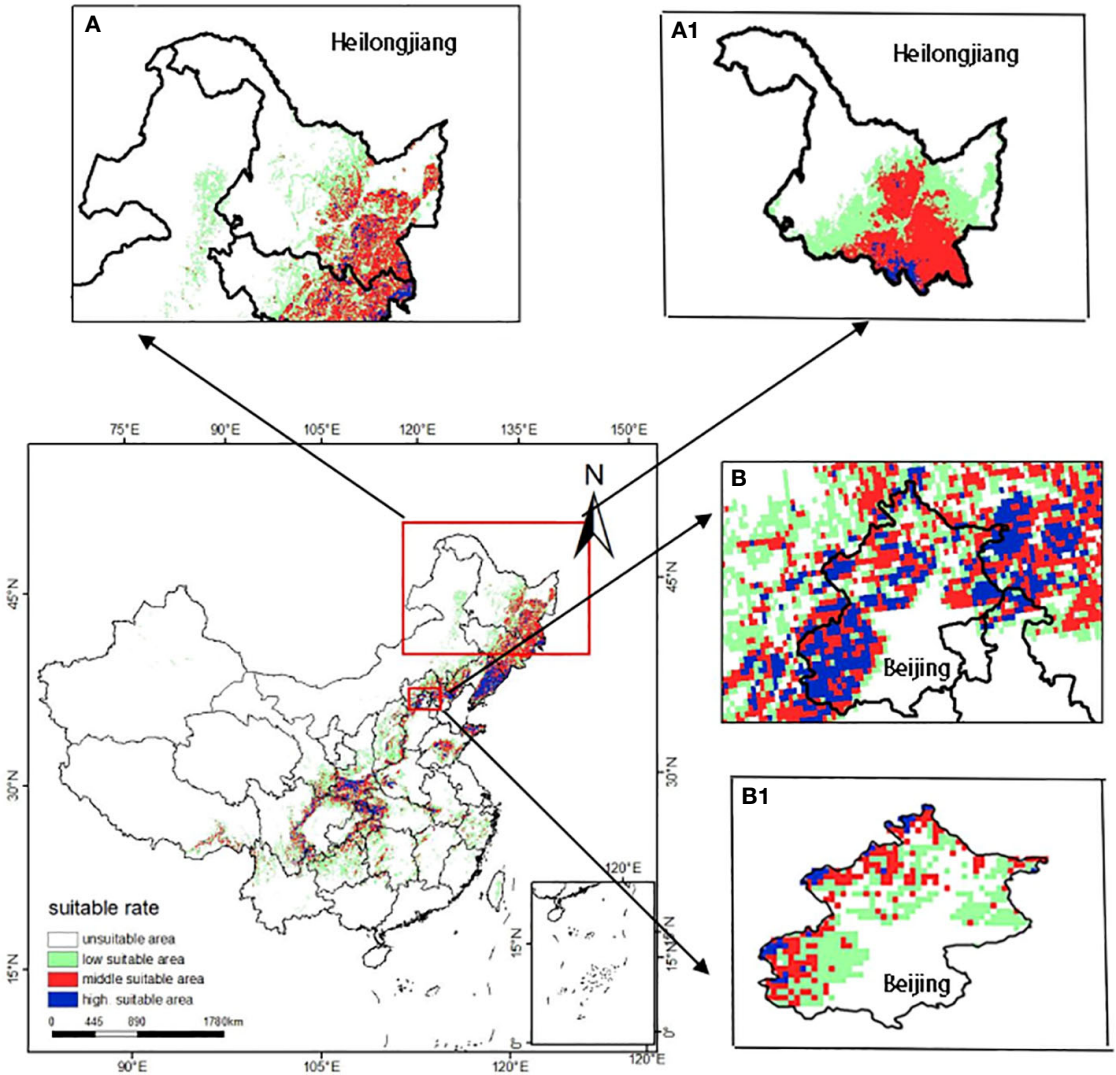
Potential distribution areas of *T. amurensis* at different administrative scales (**A, B**:national scale; **A1** :provincial scale; **B1** :local scale).

The results of the potential distribution of *T. amurensis* in Heilongjiang Province and Beijing are shown in **Figure 9**. The results show that the potential distribution of *T. amurensis* in Heilongjiang Province is basically consistent with the predicted results of *T. amurensis* at the national scale, whereas the distribution of *T. amurensis* in Beijing is inconsistent, therefore, the results of this study are only valid at the national and provincial levels, and can not be applied to local microclimates. In addition, the vector map of Beijing extracted during the analysis and validation process tends to be blurred, so the vector map of China used in this paper is not in a position to be suitable for the prediction of the potential distribution area of *T. amurensis* in smaller administrative units.

## 4 Discussion

### 4.1 Evaluation of the MaxEnt model simulation results

Among the species distribution models, the MaxEnt model is more popularly used, and the default parameters in this model are derived from earlier tests on 226 species (Phillips and Dudík, 2008). The study of species distribution under the default parameters is prone to overfitting, high complexity and may reduce the accuracy of the results. Therefore, in this study we optimized the model using the AICc parameters and adjusting the multiplicity parameter to reduce model complexity and improve the fit of predictions to the actual fit. In this study, MaxEnt was run with the optimal model parameter settings and cross-validated 10 times with an average AUC = 0.892, indicating that the model predictions were reliable. When establishing the species distribution model, some researchers only consider the climate factors. In this study, we selected climate, topography and soil factors for modelling. The results showed that the distribution of *T. amurensis* was less affected by soil factors and more affected by climate factors and topographic factors. However, there are limitations in this study, such as anthropogenic factors that may affect the distribution of *T. amurensis*. In addition, the results of this study are only valid at the national and provincial levels, and can not be applied to local microclimates.

### 4.2 Dominant environmental factors affecting potential suitable habitat of *T. amurensis*

Based on the distribution data of climate, soil, topographic environmental factors and *T. amurensis* in China, this study used MaxEnt model to evaluate the habitat suitability of *T. amurensis* in China at present and in the future.

The results showed that this model was reliable in predicting the habitat suitability of *T. amurensis*, and that bio18, slope, elev, bio1, bio9 and bio2 were the dominant environmental factors affecting the potential suitable habitat of *T. amurensis*. Du (Du, 2022) found that hydrothermal conditions were the dominant environmental factors driving the distribution pattern of woody plants in Heilongjiang basin, and the suitability distribution of *T. amurensis* was jointly affected by both temperature and precipitation, which is similar to the results of this paper. In addition to temperature and precipitation factors, the growth of *T. amurensis* is closely related to altitude and slope. Zheng (Zheng et al., 2022) found that the growth of the diameter of the chest of *T. amurensis* was relatively fast in the area of 800~900 m altitude, and the effect of slope on the growth of the diameter of the chest of this species was significant. The growth rate of *T. amurensis* was faster on mild slope and slower on steep slope. *T. amurensis * prefers fertile, well-drained moist soil and is a good landscape tree. *T. amurensis* is strongly influenced by altitude and can be used on a vertical scale to exploit its characteristics, increase its ornamental value and enhance the landscape benefits while afforestation and greening. Comparing the previous studies we found that for different woody plants, the dominant environmental factors affecting the distribution of their suitability are different. Bangken Ying (Ying et al., 2024) explored the effects of terrestrial climatic variables and ocean surface marine environmental factors on *Kandelia obovata*. The results showed that ocean surface mean water temperature, isothermality, precipitation in the warmest quarter, mean annual air temperature, and maximum temperature in the hottest month may be the main environmental factors affecting the distribution of autumn eggplant. Sun & Zhang (Sun et al., 2020) found that seasonal variations in annual precipitation and temperature were the main drivers affecting the species richness of *Quercus spp*. in China.

### 4.3 Potentially suitable habitat conditions

Understanding the potential distribution patterns of species in the context of climate change plays an important role in measuring the impacts of climate change on species and developing conservation strategies (Deb et al., 2017). The results of this study showed that the general trend of the center-of-mass of suitable habitat for *T. amurensis* will move to higher latitudes under future climate scenarios, which may be related to global warming. Some research results indicated that the range shift of organisms is unavoidable due to the earth’s warming and global climate change. For example, one study (Wang et al., 2023) show that the distribution of plants of the Chinese alpine oak group has a tendency to migrate to the low altitude and low latitudes under future climate conditions; and that the total fitness area of endangered plants Eleutharrhena macrocarpa (Diels) Forman has migrated to the direction of higher latitude since the last interglacial period (Zhao and Fan, 2021). In this study, the potential suitable areas for *T. amurensis* in the future period will be mainly distributed in Heilongjiang, Jilin and Liaoning Provinces, and the total suitable area distribution will be reduced to different degrees compared to the current period, and the habitat suitability of *T. amurensis* will decline compared to the current period, with the largest decline compared to the current period under the SSP5-8.5 climate scenario. Range shift to higher altitudes or latitudes is a major strategy for species to adapt to global warming, however, most species face a crisis of habitat loss during range shift to higher altitudes or latitudes (Williams et al., 2007), and the results of this study show that habitat suitability for *T. amurensis* in the future period will decrease, so it is particularly important to develop effective conservation measures.

The quantity and quality of *T. amurensis* populations have declined over the years as a result of extensive logging and utilization. In addition, the species has developed the characteristic of seed dormancy during phylogeny in order to adapt to the external environment. This characteristic can hinder the reproduction of *T. amurensis* which can lead to a decline in the population of the species. Therefore, the restoration of high quality populations of *T. amurensis* is urgent. At present, many scholars have carried out systematic studies on the breeding of *T. amurensis*, mainly by sowing seeds, cuttings and tissue culture. In the future, the protection of *T. amurensis* still needs efforts. On the one hand, it is necessary to strengthen the publicity, improve people’s ideological awareness, and strengthen the environmental protection and artificial forest protection of wild *T. amurensis*; On the other hand, based on the results of the study of potential suitable distribution areas for *T. amurensis* under future climate scenarios, it is particularly important to develop a long-term comprehensive conservation plan that implements the national policy guidelines and does not destroy the original land.

## 5 Conclusion

This study showed that hydrothermal conditions and topographic factors of altitude and slope were the dominant factors affecting the distribution of *T. amurensis*. The suitable habitat area of *T. amurensis* was about 14.15×10^4^ km^2^ under the current climatic conditions, accounting for 14.74% of the total area in China. In the future, under different climate scenarios, the total suitable area will reduce to different degrees compared to the current period, the distribution area of the high suitable area will increase and the distribution area of the medium and low suitable areas will decrease. The habitat suitability of *T. amurensis* will reduce, and the suitable habitat will generally move to higher latitudes. The priority protected area of *T. amurensis* was about 5.76×10^4^ km^2^, which was mainly distributed in Liaoning, Jilin, Heilongjiang, Hebei, Gansu, Shaanxi, Henan, Sichuan and Beijing. Therefore, when planning protected areas, the impact of future climate change should also be taken into account. The establishment of protected areas should be moderately shifted to higher latitudes on the basis of the current projected areas.

## Data availability statement

The raw data supporting the conclusions of this article will be made available by the authors, without undue reservation.

## Author contributions

F-GZ: Data curation, Formal analysis, Investigation, Writing – original draft. SZ: Conceptualization, Methodology, Software, Writing – review & editing. KW: Investigation, Validation, Writing – review & editing. RZ: Investigation, Validation, Writing – review & editing. GZ: Resources, Supervision, Writing – review & editing. YW: Funding acquisition, Supervision, Validation, Writing – review & editing.
